# Epidural analgesia in ICU chest trauma patients with fractured ribs: retrospective study of pain control and intubation requirements

**DOI:** 10.1186/s13613-020-00733-0

**Published:** 2020-08-27

**Authors:** Konstantinos Bachoumas, Albrice Levrat, Aurélie Le Thuaut, Stéphane Rouleau, Samuel Groyer, Hervé Dupont, Paul Rooze, Nathanael Eisenmann, Timothée Trampont, Julien Bohé, Benjamin Rieu, Jean-Charles Chakarian, Aurélie Godard, Laura Frederici, Stephanie Gélinotte, Aurélie Joret, Pascale Roques, Benoit Painvin, Christophe Leroy, Marcel Benedit, Loic Dopeux, Edouard Soum, Vlad Botoc, Muriel Fartoukh, Marie-Hélène Hausermann, Toufik Kamel, Jean Morin, Roland De Varax, Gaetan Plantefève, Alexandre Herbland, Matthieu Jabaudon, Thibault Duburcq, Christelle Simon, Russell Chabanne, Francis Schneider, Frederique Ganster, Cedric Bruel, Ahmed-Saïd Laggoune, Delphine Bregeaud, Bertrand Souweine, Jean Reignier, Jean-Baptiste Lascarrou

**Affiliations:** 1 Médecine Intensive Réanimation, District Hospital Center, La Roche-sur-Yon, France; 2 Intensive Care Unit, Regional Hospital Center, Annecy, France; 3 Plateforme de la méthodologie et de la Biostatistique, Direction de la Recherche Clinique, CHU de Nantes, 44093 Nantes Cedex, France; 4 Intensive Care Unit, Hospital Center, Angoulême, France; 5 Intensive Care Unit, Hospital Center, Montauban, France; 6 Surgical Intensive Care Unit, University Hospital, Amiens, France; 7 Surgical Intensive Care Unit, University Hospital, Nantes, France; 8 Intensive Care Unit, Jean Perrin Center, Clermont-Ferrand, France; 9 Intensive Care Unit, University Hospital, Limoges, France; 10 University Hospital, Lyon Sud, Lyon, France; 11 Surgical Intensive Care Unit, Gabriel-Montpied University Hospital, Clermont-Ferrand, France; 12 Intensive Care Unit, Regional Hospital Center, Roanne, France; 13 Intensive Care Unit, Regional Hospital Center, Saint-Brieuc, France; 14 Intensive Care Unit, Regional Hospital Center, Sud Francilien, Corbeil-Essone, France; 15 Intensive Care Unit, Regional Hospital Center, Dieppe, France; 16 Surgical Intensive Care Unit, University Hospital, Caen, France; 17 Intensive Care Unit, Regional Hospital Center, Cherbourg, France; 18 Intensive Care Unit, Regional Hospital Center, Lorient, France; 19 Intensive Care Unit, Regional Hospital Center, Puy en Velay, France; 20 Intensive Care Unit, Regional Hospital Center, Moulins, France; 21 Intensive Care Unit, Regional Hospital Center, Vichy, France; 22 Intensive Care Unit, Regional Hospital Center, Périgueux, France; 23 Intensive Care Unit, Regional Hospital Center, Saint-Malo, France; 24 Intensive Care Unit, University Hospital, Tenon, Paris, France; 25 Intensive Care Unit, Regional Hospital Center, Aurillac, France; 26 Intensive Care Unit, Regional Hospital Center, Orléans, France; 27 Respiratory Care Unit, University Hospital, Nantes, France; 28 Intensive Care Unit, Regional Hospital Center, Macon, France; 29 Intensive Care Unit, Regional Hospital Center, Argenteuil, France; 30 Intensive Care Unit, Regional Hospital Center, La Rochelle, France; 31 Department of Perioperative Medicine, CHU Clermont-Ferrand and GReD, CNRS, UMR 6293, INSERM U1103, Universite Clermont Auvergne, Clermont-Ferrand, France; 32 Medical Intensive Care Unit, University Hospital, Lille, France; 33 Intensive Care Unit, Regional Hospital Center, Versailles, France; 34 Neurological Intensive Care Unit, University Hospital, Clermont-Ferrand, France; 35 Médecine Intensive-Réanimation, Hôpital de Hautepierre, Hôpitaux Universitaires de Strasbourg, Strasbourg, France; 36 Intensive Care Unit, Regional Hospital Center, Mulhouse, France; 37 Intensive Care Unit, Saint-Joseph Hospital Center, Paris, France; 38 Intensive Care Unit, Regional Hospital Center, Guéret, France; 39 Intensive Care Unit, Regional Hospital Center, Châteauroux, France; 40 Medical Intensive Care Unit, Gabriel-Montpied University Hospital, Clermont-Ferrand, France; 41 Médecine Intensive Réanimation, University Hospital, Nantes, France

**Keywords:** Epidural analgesia, Chest trauma

## Abstract

**Background:**

Nonintubated chest trauma patients with fractured ribs admitted to the intensive care unit (ICU) are at risk for complications and may require invasive ventilation at some point. Effective pain control is essential. We assessed whether epidural analgesia (EA) in patients with fractured ribs who were not intubated at ICU admission decreased the need for invasive mechanical ventilation (IMV). We also looked for risk factors for IMV.

**Study design and methods:**

This retrospective, observational, multicenter study conducted in 40 ICUs in France included consecutive patients with three or more fractured ribs who were not intubated at admission between July 2013 and July 2015.

**Results:**

Of the 974 study patients, 788 were included in the analysis of intubation predictors. EA was used in 130 (16.5%) patients, and 65 (8.2%) patients required IMV. Factors independently associated with IMV were chronic respiratory disease (*P *= 0.008), worse SAPS II (*P *< 0.0001), flail chest (*P *= 0.02), worse Injury Severity Score (*P *= 0.0003), higher respiratory rate at admission (*P *= 0.02), alcohol withdrawal syndrome (*P *< 0.001), and noninvasive ventilation (*P *= 0.04). EA was not associated with decreases in IMV requirements, median numerical rating scale pain score, or intravenous morphine requirements from day 1 to day 7.

**Conclusions:**

EA was not associated with a lower risk of IMV in chest trauma patients with at least 3 fractured ribs, moderate pain, and no intubation on admission. Further studies are needed to clarify the optimal pain control strategy in chest trauma patients admitted to the ICU, notably those with severe pain or high opioid requirements.

## Take home points

Study question: Does epidural analgesia improve pain control and/or decrease intubation requirements in patients with at least three fractured ribs who are not intubated at ICU admission?

Results: The proportions of patients requiring intubation, pain scores, and intravenous analgesic needs were not significantly different in patients with vs. without epidural analgesia.

Interpretation: Epidural analgesia was not associated with a lower risk of invasive mechanical ventilation in chest trauma patients with at least 3 fractured ribs, moderate pain, and no intubation on admission.

## Introduction

Fractured ribs are the most common chest injuries, being present in about 10% of trauma patients [1, 2]. In the past, invasive mechanical ventilation (IMV) was the reference standard treatment for patients with multiple fractured ribs [3]. Whereas the need for IMV has decreased over time [4], mortality rates have remained remarkably stable in recent years [4, 5], with high mortality in patients with severe trauma [6], notably those who also have extrathoracic injuries [7]. These patients are usually intubated before intensive care unit (ICU) admission. Patients with fractured ribs who are not intubated at ICU admission may require IMV during their ICU stay and are at risk for several complications including pneumonia, atelectasis, and acute respiratory distress syndrome [8–11]. Older age, greater number of fractured ribs, concomitant injuries, and chronic lung disease are the main documented risk factors for complications [12, 13].

Achieving effective pain control is a key goal in the treatment of chest injuries [14]. Pain limits coughing efficiency and secretion clearance, thereby potentially leading to progressive atelectasis, loss of functional residual capacity (FRC) and, ultimately, respiratory distress. Pain control can improve ventilatory function and prevent respiratory complications [15]. Multiple modalities are available for alleviating pain due to chest injuries. Guidelines recommend epidural analgesia over nonregional modalities of pain control, but rest on a very low level of evidence [16, 17]. No randomized controlled trial (RCT) has evaluated whether EA decreases the need for IMV in patients with chest injuries. A recent meta-analysis concluded that EA was not associated with a shorter IMV duration [18], whereas an analysis of a large dataset showed an association between EA and reduced mortality [19]. In addition, EA is performed only in 10–15% of patients with fractured ribs [20], due to the existence in some patients of contraindications and to the need for specially trained staff to ensure safe catheter insertion and analgesic administration. We hypothesized that recent improvements in EA modalities might translate into better pain control with fewer technical obstacles, leading to an improvement in respiratory function with decreased IMV needs.

The primary aim of this retrospective study was to assess whether EA decreased IMV needs in chest trauma patients with fractured ribs who were not intubated at ICU admission. We also looked for factors predicting a need for IMV, under the hypothesis that knowledge of such factors might, in the future, improve the identification of those patients likely to benefit from EA.

## Methods

### Study design and patients

We conducted a retrospective, observational, multicenter study in 40 ICUs in France affiliated to 24 regional hospitals and 10 teaching hospitals.

The inclusion criteria were three or more posttraumatic fractured ribs [21], age > 18 years, ICU admission between July 2013 and July 2015, and spontaneous breathing at ICU admission (including non-invasive ventilation). Noninclusion criteria were IMV started before ICU admission and previous ICU stays during the same hospital stay.

Eligible patients were identified by searching the electronic databases of each participating hospital using the International Classification of Diseases, 10th revision codes S22.4 for multiple fractured ribs, S22.5 for flail chest, and S27.1 for traumatic hemothorax. All patients with these codes were screened, and those meeting all the inclusion criteria and none of the noninclusion criteria were enrolled in the study.

### Definitions

Flail chest was defined as fractures of three or more consecutive ribs in two or more places resulting in paradoxical chest wall motion during breathing [21]. Pneumonia was defined as early-onset pneumonia if onset occurred within 48 h after ICU admission and as nosocomial pneumonia if onset occurred later during the ICU stay. Patients were considered as having alcohol withdrawal syndrome if this diagnosis was recorded in the medical file by the physician in charge of the patient, before intubation if IMV was eventually required [22]. The numerical rating scale (NRS) scores for pain were recorded; on this scale, 0 indicates no pain and 10 the worst possible pain. Contraindications of EA were coagulation disorders (platelets < 50,000/mm^3^, prothrombin time < 50%); treatment with ticlopidine, clopidogrel, prasugrel, ticagrelor, or one of the new anticoagulants; unstable spinal fracture; impaired consciousness or agitation; acute respiratory failure requiring immediate IMV; and shock requiring vasoactive drug administration [23].

### Data collection

At each ICU, a local investigator abstracted the following data from the paper and/or electronic files of each patient: age, sex, Knaus and McCabe scores, and comorbidities (respiratory disease, smoking, alcohol abuse, obesity defined as a body mass index > 30); respiratory rate, oxygen saturation, oxygen flow at ICU admission (first values in the patient file), and PaO_2_/FiO_2_ ratio calculated from SpO_2_/FiO_2_ using the formula from Rice et al. [24], with FiO_2_ estimated according to Coudroy et al. [25]; times from trauma to emergency department arrival and to ICU admission; number of fractured ribs; and presence of flail chest. The initial computed tomography (CT) scan was reviewed and the Injury Severity Score (ISS) and Abbreviated Injury Scale (AIS) score for each body region were determined by the same principal investigator to ensure reliability. The 2008 update of the 2005 AIS was used [26]. The following data from the ICU stay were also collected: Simplified Acute Physiology Score II (SAPS II) [27]; chest tube insertion during the ICU stay; NRS scores during the first 7 days; intravenous morphine consumption during the first 24 h (mg); need for intravenous morphine during the first 7 days (if other step-3 analgesics were used intravenously, their doses were converted to morphine equivalents); use of other analgesics including nonsteroidal antiinflammatory drugs (NSAIDs), ketamine, tramadol, paracetamol, and nefopam; use of EA (the criteria for using EA were at the discretion of the physician in charge and were not recorded in the case report form); times from hospital and ICU admissions to EA; EA duration (days); EA modalities; complications of EA (epidural hematoma, epidural abscess, severe hypotension); contraindications of EA; patient refusal of EA; use of intercostal analgesia, with the time and duration if used; use of IMV with the reasons for IMV (respiratory, circulatory, and/or neurological failure; need for emergent surgery); time of intubation and duration of IMV; use of bilevel noninvasive ventilation (NIV) (before intubation if IMV was needed), with the reason (hypercapnic or pure hypoxic respiratory failure) and duration of NIV; surgical rib stabilization occurrence of alcohol withdrawal syndrome (before intubation if IMV was needed); occurrence of early-onset or nosocomial pneumonia; occurrence of ventilator-associated pneumonia (VAP); ICU and hospital stay lengths; and ICU and hospital mortality.

### Statistical analysis

Qualitative data are described as counts and percentages and quantitative data as mean ± SD if normally distributed and as median [IQR] otherwise. We checked the linearity of quantitative variables using the Shapiro–Wilk test. Nonlinear continuous variables were dichotomized based on their median.

We confined the analysis to patients without emergent surgery, contraindications of EA, failure to insert the epidural catheter, and refusal to receive EA. Univariate analyses were performed to identify factors associated with IMV. The Fine–Gray competing risks regression model was used to estimate subdistribution hazard ratios with their 95% confidence intervals (95% CIs). A multivariate model for predicting the need for IMV was then constructed using the variables associated with IMV at *P* values ≤ 0.2 by univariate analysis. Backward selection was applied until all remaining variables met the 0.05 threshold in the multivariate model. Only patients with no missing data were included in the multivariate analysis. Finally, we repeated the multivariate analysis in patients from ICUs where EA was used in at least 1 study patient and in patients whose NRS pain score was > 3 on day 1.

All analyses were two-sided, and *P* values < 0.05 were considered significant. SAS software (v. 9.4 for Windows; SAS Institute) was used for the statistical analyses.

## Results

### Patient features at ICU admission and outcomes (Table 1 and Additional file 1: Figure S1)

We included 974 patients from 40 ICUs. Table 1 reports their main features and outcomes. IMV was required in 128 (13.1%) patients.

**Table 1:** Baseline characteristics and outcomes

	974 patients	Missing data
Demographic variables at baseline		
Age, years, mean ± SD	57 ± 17	10
Males, *n* (%)	744/971 (76.6%)	3
Comorbidities, *n* (%)		
McCabe score		57
(0) No fatal underlying disease	812/917 (88.5%)	
(1) Death expected within 5 years	96/917 (10.5%)	
(2) Death expected within 1 year	9/917 (1%)	
History of respiratory disease	130/969 (13.4%)	5
COPD	68/119 (57%)	11
Obstructive sleep apnea	31/108 (28.7%)	22
Asthma	25/111 (22.5%)	19
Pulmonary fibrosis	6/105 (5.7%)	25
Lung cancer	2/108 (1.8%)	22
Other	16/109 (14.6%)	21
History of alcohol abuse	173/948 (18.2%)	26
History of smoking	342/930 (36.7%)	44
Obesity (BMI ≥ 30)	132/956 (13.8%)	18
Characteristics of the injury		
Number of fractured ribs, mean ± SD	6 ± 3	7
Flail chest, *n* (%)	245/957 (25.6%)	17
Isolated chest trauma, *n* (%)	276/971 (28.4%)	3
PaO_2_/FiO_2_ ratio at ICU admission^a^	320 [254–390]	25
SAPS II, mean ± SD	22 ± 11	26
Injury Severity Score, mean ± SD	18 ± 7	0
Thoracic AIS ≥ 3, *n* (%)	928 (5.3%)	0
Head AIS ≥ 3, *n* (%)	82 (8.5%)	6
Abdominal AIS ≥ 3, *n* (%)	97 (10.0%)	7
Face AIS ≥ 3, *n* (%)	1 (0.10%)	9
Extremity AIS ≥ 3, *n* (%)	82 (8.66%)	4
External AIS ≥ 3, *n* (%)	0 (0%)	12
Time from injury to hospital admission, days, median [IQR]	0 [0–0]	3
Time from injury to ICU admission, days, median [IQR]	0 [0–1]	7
Outcome		
IMV, *n* (%)	128/974 (13.1%)	
Time from hospital admission to IMV, days, median [IQR]	2 [1–4]	1
Reason for IMV (some patients had more than one reason)		
Acute respiratory failure	99	
Acute circulatory failure	10	
Acute neurologic disorder	14	
Emergency surgery	21	
Time from ICU admission to IMV, days, median [IQR]	2 [1–3]	1
IMV duration, days, median [IQR]	8 [3–14]	3
Bilevel NIV, *n* (%)	268/961 (27.9%)	13
Reason for NIV		13
Hypercapnia	82 (32.1%)	
Hypoxemia	165 (64.8%)	
Prophylactic	8 (3.1%)	
NIV duration, days, median [IQR]	3 [1–4]	
Chest tube insertion, *n* (%)	429/948 (45.2%)	26
Hemothorax	146/425 (34%)	4
Pneumothorax	159/425 (37%)	4
Hemothorax and pneumothorax	119/425 (28%)	4
Surgery	1/425 (0.2%)	4
Surgical stabilization of ribs, *n* (%)	44/962(4.5%)	12
Alcohol withdrawal syndrome, *n* (%)	46/956 (4.8%)	18
Infection, *n* (%)		
Early-onset pneumonia (within 48 h after ICU admission)	89/954 (9.3%)	20
Nosocomial pneumonia (> 48 h after ICU admission)	89/912 (9.8%)^b^	26
ICU stay length, median [IQR]	5 [3–9]	6
Length of hospital stay (median, IQR)	11 [7–18]	19
ICU mortality (*N*, %)	31/965 (3.2%)	9
Hospital mortality (*N* %)	37/958 (3.8%)	16

*MD* missing data, *COPD* chronic obstructive pulmonary disease, *BMI* body mass index, *IQR* interquartile range, *IMV* invasive mechanical ventilation, *AIS* Abbreviated Injury Scale, *SAPS II* Simplified Acute Physiologic Score II, *ICU* intensive care unit, *NIV* noninvasive ventilation

^a^ PaO_2_/FiO_2_ was not collected but was derived from SpO_2_ at ICU admission and the oxygen flow rate

^b^ 36 patients died or were discharged within 48 h after ICU admission

### Pain management and use of epidural analgesia (EA) (Table 2, Additional file 1: Figure S1 and Additional file 2: Figure S2)

EA was used in 130/788 (16.5%) patients (Table 2). Of the 974 patients included, 21 required emergent surgery, 153 had contraindications, 4 refused EA, 6 had failed catheter insertion, and 2 had no data on EA use (Additional file 1: Figure S1). The proportion of patients who received EA varied widely across study ICUs, from 0 to 62% (Additional file 2: Figure S2). The only complication was severe hypotension with cardiac arrest in 1 patient. EA had no pain-relieving effect in 5 (4%) patients.

**Table 2:** Pain management and use of epidural analgesia

	No epidural analgesia (*N* = 658)	Epidural analgesia (*N* = 130)	*P* value
Baseline characteristics			
Age, years, mean ± SD	55 ± 17^a^	57 ± 16^b^	0.15
History of chronic respiratory disease, *n* (%)	83/653 (12.7%)	14/130 (10.8%)	0.54
History of alcoholism, *n* (%)	105/640 (16.4%)	26/123 (21.1%)	0.20
History of smoking, *n* (%)	227/633 (35.9%)	44/123 (35.8%)	0.98
Obesity (BMI ≥ 30), *n* (%)	83/648 (12.8%)	25/126 (19.8%)	0.04
Number of fractured ribs ≥ 6, *n* (%)	312/656 (47.6%)	87/129 (67.4%)	< 0.0001
Flail chest, *n* (%)	143/648 (22.1%)	57/129 (44.2%)	< 0.0001
Thoracic AIS (mean ± SD)	3.46 ± 0.61	3.75 ± 0.53	< 0.0001
Injury Severity Score, mean ± SD	17.5 ± 7.0	20.2 ± 7.0	0.0001
SAPS II, mean ± SD	21 ± 10^c^	21 ± 9^d^	0.46
Clinical variables at ICU admission			
Oxygen saturation, %, mean ± SD	96 ± 5^e^	96 ± 3^f^	0.81
Respiratory rate, breaths/min, mean ± SD	20 ± 5 ^g^	20 ± 5 ^h^	0.87
Oxygen flow > 6 L/min, *n* (%)	117/649 (18.0%)	37/127 (29.1%)	0.004
History of alcoholism			
No	535^i^	97^j^	0.41
Yes, without symptoms of withdrawal	80	19	
Yes, with symptoms of withdrawal	25	7	
Outcomes			
Median NRS pain score on day 1 > 4, *n* (%)	243/637 (38.1%)	60/125 (48.0%)	0.04
Maximum NRS pain score from day 1 to day 4, median [IQR]	6 [5.0–8.0]	7 [5.0–8.0]	0.03
Median numerical rating scale pain score, median [IQR]			
Day 1	3.0 [1.0–5.0]^k^	3.5 [1.0–5.0]^l^	0.23
Day 2	2.5 [1.0–4.0]^k^	2.5 [0.5–4.0]^l^	0.37
Day 3	2.0 [0.0–4.0]^k^	2.0 [0.0–3.0]^l^	0.49
Day 4	2.0 [0.0–3.5]^k^	1.0 [0.0–3.0]^l^	0.01
Day 5	2.0 [0.0–3.5]^k^	1.0 [0.0–3.0]^l^	0.09
Day 6	2.0 [0.0–3.0]^k^	2.0 [0.0–3.0]^l^	0.36
Day 7	1.5 [0.0–3.0]^k^	2.0 [0.0–3.5]^l^	0.64
Intravenous morphine			
Day 1, *n* (%)	395/657 (60.1%)	76/130 (58.5%)	0.72
Dose during the first 24 h in the ICU, mg, median [IQR]	5 [0–16]^m^	5.0 [0.0–14]^n^	0.78
Day 7, *n* (%)	62/657 (9.4%)	27/130 (20.8%)	0.0002
Other analgesics during the first 7 ICU days, *n* (%)			
NSAIDs	186/629 (29.6%)	42/129 (32.6%)	0.50
Ketamine	63/625 (10.1%)	18/127 (14.2%)	0.17
Nefopam	478/639 (74.8%)	96/128 (75.0%)	0.96
Paracetamol	625/654 (95.6%)	124/129 (96.1%)	0.78
Tramadol	361/633 (57.0%)	69/129 (53.5%)	0.46
Intercostal anesthesia	18/658 (2.1%)		
Days with intercostal anesthesia, median [IQR]	4 [3–5.5]		
Epidural analgesia			
Patients with EA, *n* (%)		130/788 (16.5%)	
Time from hospital admission to EA, days, median [IQR]		1 [0–2]	
Time from ICU admission to EA, days, median [IQR]		1 [0–2]	
EA duration, days, mean ± SD		4.1 ± 2.2^o^	
Local anesthetics, *n* (%)			
Ropivacaine		118/124 (95.2%)	
Bupivacaine		0/124 (0%)	
Levobupivacaine		6/124 (4.8%)	
Local opioids, *n* (%)			
Sufentanil		96/125 (76.8%)	
Morphine		7/125 (5.6%)	
None		22/125 (17.6%)	
Complications, *n* (%)			
Epidural hematoma		0	
Epidural abscess		0	
Severe hypotension		1	
No pain relief from EA, *n* (%)		5/111 (4.5%)	
Mechanical ventilation			
IMV, *n* (%)	53/658 (8.0%)	12/130 (9.2%)	0.65
Time from ICU admission to IMV, days, median [IQR]	2 [1–4]^p^	2.5 [1.5–5]^q^	0.52
IMV duration, days, median [IQR]	9.5 [5–16]^p^	10 [4.5–13]^q^	0.97
Reason of IMV initiation (more than one possible for each patient)			
Acute respiratory failure	51	10	0.15
Acute neurologic failure	6	3	0.35
Acute circulatory failure	3	1	0.57
Noninvasive ventilation, *n* (%)	146/647 (22.6%)	58/129 (45.0%)	< 0.0001
ICU stay length, days, median [IQR]	5 [2–8]^r^	8 [6–12]	< 0.0001
Hospital stay length, days, median [IQR]	10 [6–17]^s^	13 [9–18]^t^	0.0002
ICU mortality, *n* (%)	13/653 (2.0%)	5/129 (3.9%)	0.20
Hospital mortality, *n* (%)	15/650 (2.3%)	5/127(3.9%)	0.35

Where the available number of patients with data is not specified, all patients in the group had available data

*BMI* body mass index, *NRS* numerical rating scale, *IQR* interquartile range, *AIS* Abbreviated Injury Scale, *ISS* Injury Severity Score, *SAPS II* Simplified Acute Physiology Score version II, *NSAIDs* nonsteroidal anti-inflammatory drugs, *EA* epidural analgesia, *IMV* invasive mechanical ventilation

^a^ Available for 652/658 patients

^b^ Available for 128/130 patients

^c^ Available for 646/658 patients

^d^ Available for 124/130 patients

^e^ Available for 650/658 patients

^f^ Available for 128/130 patients

^g^ Available for 635/658 patients

^h^ Available for 123/130 patients

^i^ Available for 622/658 patients

^j^ Available for 116/130 patients

^k^ Available on days 1 through 7 for 637, 598, 505, 408, 300, 232, and 170 patients, respectively

^l^ Available on days 1 through 7 for 125, 123, 121, 116, 105, 96, and 71 patients, respectively

^m^ Available for 642/658 patients

^n^ Available for 129/130 patients

^o^ Available for 129/130 patients

^p^ Available for 52/53 patients

^q^ Available for 12/12 patients

^r^ Available for 654/658 patients

^s^ Available for 650/658 patients

^t^ Available for 128/130 patients

Compared to the group without EA, the group with EA had higher mean values for the ISS and thoracic AIS score, a larger number of fractured ribs, a higher proportion of patients with flail chest, and greater oxygen needs at ICU admission. However, the EA group was similar to the non-EA group regarding the median NRS pain score and need for intravenous morphine during the first 7 ICU days. Also, the need for IMV was not significantly different between the two groups. The time from ICU admission to IMV initiation was not significantly shorter in the group without EA. NIV use was significantly more common in the EA group, which had longer median ICU and hospital stays compared to the non-EA group.

### Risk factors for invasive mechanical ventilation (IMV) (Additionnal file 3: Table S1 and Additionnal file 4: Table S2, Tables 3, 4)

**Fig. 1:**
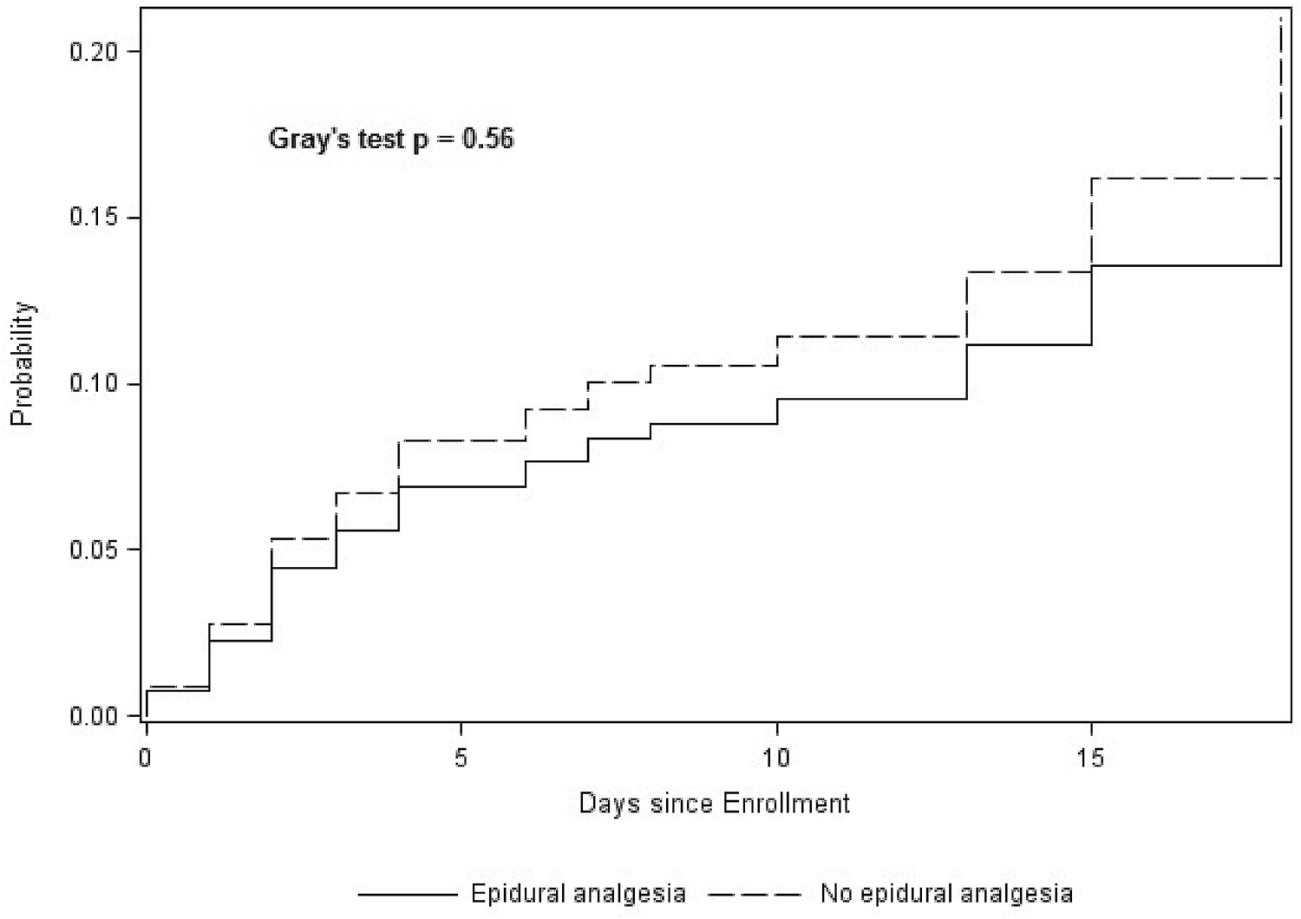
Cumulative incidence of invasive mechanical ventilation

IMV was required in 65 (8.2%) patients. Table 3 reports the factors significantly associated with requiring IMV by univariate analysis. Figure 1 shows the cumulative incidence of IMV over time in the groups with and without EA.

**Table 3:** Characteristics and outcomes of patients with and without invasive mechanical ventilation (IMV); patients who received IMV for emergent surgery, with contraindication for EA, refused EA, failed catheter insertion, missing data about EA were excluded

	IMV (*N* = 65)	No IMV (*N* = 723)	HR (95% CI)	*P* value
Patient characteristics				
Age, years, mean ± SD	62 ± 15	55 ± 17	1.02 (1.01–1.04)	0.020
Chronic respiratory disease, *n* (%)	23/65 (35.4%)	74/718 (10.3%)	3.98 (2.39–6.61)	< 0.001
Obesity (BMI > 30), *n* (%)	12/64 (18.7%)	96/710 (13.5%)	1.38 (0.72–2.61)	0.33
History of smoking, *n* (%)	32/64 (50.0%)	239/692 (34.5%)	1.91 (1.17–3.10)	0.009
Alcohol, *n* (%)				< 0.001*
No chronic alcoholism	43/64 (67.2%)	589/699 (84.3%)	1 (reference)	
Alcohol abuse without alcohol withdrawal syndrome	10/64 (15.6%)	89/699 (12.7%)	1.64 (0.83–3.27)	
Alcohol abuse with alcohol withdrawal syndrome	12/64 (17.2%)	21/699 (3.0%)	5.14 (2.54–10.4)	
Injury characteristics				
Thoracic AIS, mean ± SD	3.8 ± 0.6	3.5 ± 0.6	2.02 (1.18–3.47)	0.01
ISS, mean ± SD	21 ± 8	18 ± 7	1.05 (1.01–1.08)	0.006
Flail chest, *n* (%)	28/65 (43.1%)	172/712 (24.2%)	2.01 (1.24–3.28)	0.005
≥ 6 fractured ribs, *n* (%)	36/64 (56.2%)	363/721 (50.3%)	1.13 (0.68–1.86)	0.64
SAPS II, mean ± SD	30.6 ± 14^b^	20 ± 9^c^	1.08 (1.06–1.09)	< 0.001
Clinical variables				
Oxygen saturation at ICU admission, %, mean ± SD	95 ± 4	96 ± 5^d^	0.98 (0.96–1.00)	0.03
Respiratory rate at ICU admission, breaths/min, mean ± SD	23.0 ± 6.8^e^	20.4 ± 5.5^f^	1.07 (1.03–1.12)	0.002
Oxygen flow > 6 L/min at ICU admission, *n* (%)	28/64 (43.7%)	126/712 (17.7%)	3.08 (1.87–5.05)	< 0.001
Median NRS pain score on day 1 > 4	31/61 (50.8%)	272/701 (38.8%)	1.49 (0.91–2.43)	0.12
Treatment				
Epidural analgesia, *n* (%)	12/65 (18.5%)	118/723 (16.3%)	0.83 (0.43–1.57)	0.56
Noninvasive ventilation, *n* (%)	36/64 (56.2%)	168/712 (23.6%)	3.05 (1.88–4.97)	0.005

Where the available number of patients with data is not specified, all patients in the group had available data

*BMI* body mass index, *AIS* Abbreviated Injury Scale, *ISS* Injury Severity Score, *SAPS II* Simplified Acute Physiology Score version II, *NRS* numerical rating scale

^a^ Available for 715/723 patients

^b^ Available for 63/65 patients

^c^ Available for 707/723 patients

^d^ Available for 713/723 patients

^e^ Available for 58/65 patients

^f^ Available for 700/723 patients

* Global value

**Table 4:** Multivariate analysis to identify risk factors associated with invasive mechanical ventilation (IMV) in the 695 patients with no contraindications to, refusal of, or failure of catheter insertion for epidural analgesia

	aHR	95% CI	*P* value
History of chronic respiratory disease	2.38	1.25–4.52	0.008
SAPS II	1.07	1.04–1.09	< 0.0001
Flail chest	2.00	1.13–3.52	0.02
ISS	1.08	1.04–1.12	< 0.0001
Respiratory rate, breaths/min	1.06	1.01–1.12	0.02
Epidural analgesia	0.52	0.25–1.08	0.08
Noninvasive ventilation	1.84	1.02–3.33	0.04
Alcohol			
No alcohol abuse			< 0.0001*
Alcohol abuse without alcohol withdrawal syndrome	2.82	1.38–5.77	.
Alcohol abuse with alcohol withdrawal syndrome	7.50	3.61–15.6	

*aHR* adjusted hazard ratio, *95% CI* 95% confidence interval, *SAPS II* Simplified Acute Physiology Score version II, *ISS* Injury Severity Score

* Global value

Table 4 reports the results of the multivariate analysis in the 695 patients with no contraindications to, refusal of, or failure of catheter insertion for EA. Factors independently associated with requiring IMV were chronic respiratory disease, worse SAPS II, flail chest, worse ISS, higher respiratory rate at ICU admission, alcohol withdrawal syndrome, and prior use of NIV. EA was not independently associated with a decreased need for IMV.

The Additional file 3: Table S1 and Additional file 4: Table S2 show the results of the sensitivity analyses restricted to ICUs where at least 1 patient received EA and to patients whose NRS pain score was > 3 on day 1, respectively. In neither sensitivity analysis was EA associated with a decreased need for IMV (Additional file 3: Table S1 and Additional file 4: Table S2).

## Discussion

In our large retrospective multicenter cohort study of chest trauma patients, EA was not associated with decreased IMV use. Factors independently associated with IMV were chronic respiratory disease, worse SAPS II, flail chest, worse ISS, higher respiratory rate at ICU admission, and chronic alcohol abuse with alcohol withdrawal syndrome.

Pain control is a crucial component of the treatment strategy for patients with fractured ribs. Multiple fractured ribs cause severe pain that adversely affects the ability to cough and breathe deeply, thereby increasing the risk of secretion build-up and respiratory failure. In adults with blunt chest trauma, EA is recommended over nonregional pain-control modalities (i.e., intravenous or enteral analgesics such as opioids, acetaminophen, and NSAIDs) [16]. However, this recommendation is based on low-quality evidence, explaining perhaps in part the low compliance rates of 10% to 18% in several studies [20, 28, 29]. Similarly, in our study, EA was administered to only 16.5% of patients, and this proportion varied widely, from 0 to 62%, across study ICUs. In 658 patients, no reason for not performing EA was recorded in the file. The disadvantages of EA may explain the low utilization rate. Catheter insertion may be technically demanding, and contraindications may be present in vulnerable patients admitted to the ICU. In our study, the physician in charge determined that EA was contraindicated in 153 (16%) patients, and catheter insertion failed in 6 (1%) additional patients. Complications of EA include hypotension, epidural hematoma [30], epidural infection [31], nausea, vomiting, pruritus, respiratory depression, and urinary retention [14]. EA can increase venous pooling, thereby increasing the risk of deep vein thrombosis [32]. However, serious complications are rare [33]. In our study, no patient experienced epidural hematoma or infection, and a single patient had severe hypotension with cardiac arrest very shortly after catheter insertion. However, this last patient had respiratory failure and hypotension before EA initiation. Conceivably, the use of more peripheral regional anesthesia approaches might provide better pain control than EA. Examples include the posterior paramedian sub rhomboidal block [34] and the erector spinae plane block [35]. However, data on these two methods are still scarce.

Nevertheless, no RCT has assessed the efficacy of EA in reducing the need for IMV. In three observational studies, EA was associated with increased IMV requirements [15, 20, 36]. However, these studies also included patients who were intubated before hospital admission. EA decreased the number of ventilator days in two RCTs [21, 37], but the sample sizes were small and the studies were mostly conducted before the recent major advances in IMV use and in weaning off IMV. Pulmonary complications were assessed in a few studies but varied widely, perhaps in part due to the diversity of definitions used [21, 38]. In several studies, EA was associated with longer hospital stays, suggesting inappropriate use of this analgesic modality [39]. In three meta-analyses, EA did not significantly affect IMV duration or lengths of ICU or hospital stay [18, 33, 40]. In a database study, Malekpour et al. [19] used propensity score matching to compare EA to a paravertebral block and found no difference regarding in-hospital mortality, IMV use or duration, ICU admission, or length of stay. When patients who had either procedure were pooled and compared to patients who had neither procedure, having a procedure was associated with more ICU admissions and longer stays, but having no procedure was associated with greater mortality. In our study of patients with at least three fractured ribs who were not intubated at ICU admission, EA was not associated with decreased IMV requirements.

The associations of EA with worse ISS and thoracic AIS score values and with a larger number of fractured ribs suggest that greater injury severity was a criterion used by physicians to select patients for EA. However, our multivariate analysis excluding patients with contraindications to EA or failure of EA catheter insertion, as well as those who refused EA, showed no significant association of EA with a decreased need for IMV. This result was replicated in our sensitivity analysis confined to ICUs where at least 1 study patient received EA. Furthermore, EA was not associated with improved pain control: the groups with and without EA had similar NRS pain scores and intravenous morphine doses over the first 7 ICU days. In three RCTs assessing the efficacy of EA, pain was decreased during coughing and deep breathing, but was not significantly alleviated at rest [38, 41, 42].

Another possible explanation to the inability of EA to decrease IMV requirements or improve pain control may be failure to identify those patients most likely to benefit from EA. A retrospective study suggested that EA started early after chest trauma was not associated with a lower incidence of pulmonary complications or with shorter ICU or hospital stay lengths [28]. Conceivably, the patients most likely to benefit from EA might be those with risk factors for IMV or ARDS [43, 44]. Pain is a known major risk factor for requiring IMV [45]. Many of our patients had low morphine requirements at the time of EA initiation. Several risk factors for IMV identified in our study have been reported previously, such as variables reflecting the severity of the chest trauma, the presence of an underlying respiratory disease, and the severity of respiratory impairment at ICU admission [12]. An RCT assessing the effects of EA started within 24 h after the ICU admission of nonintubated chest trauma patients would be expected to provide a sufficiently high level of evidence to allow the development of guidelines.

Our study has several limitations. First, many confounders may have affected our results. The proportion of patients who received EA varied widely across the study ICUs, suggesting marked differences in indications. The indications for starting IMV and the ventilation protocols may also have varied according to local practice. We did not collect several variables of interest such as details on physiotherapy or the occurrence of pain during physiotherapy. Nevertheless, physiotherapy was provided locally according to specific French guidelines for chest trauma patients [17]. Alcohol withdrawal syndrome was a major risk factor for IMV, in keeping with previous data [46]. However, only 4.8% of patients experienced alcohol withdrawal syndrome and, of the 128 patients who required IMV, only 14 were intubated for neurological reasons. Finally, the number of recruited patients was also extremely variable from one ICU to the next. Second, limitations inherent in the retrospective study design include the existence of missing data and the risk of errors in data abstraction. However, missing data accounted for no more than 5% of the total. Third, reliable data were unavailable for the use of high-flow nasal oxygen therapy and for several complications. We were thus unable to assess potential associations of EA with deep vein thrombosis and with pulmonary embolism, which were reported in other studies [32]. Several complications of EA such as nausea, vomiting, and mild hypotension were not collected. Finally, patients who received IMV because they required emergent surgery were excluded from the analysis. These patients may have received EA or developed respiratory complications after surgery. A major strength of our study is the large number of patients and participating centers, however our study can be underpowered to detect clinically relevant improvement associated with EA.

## Interpretation

In our large retrospective analysis of 974 patients conducted in 40 ICUs, EA was not associated with decreased IMV needs in chest trauma patients with three or more fractured ribs who were not intubated at ICU admission. EA might, however, have benefits in the subgroup of patients with risk factors for IMV and in patients with severe pain requiring high doses of opioids and/or co-analgesics. RCTs are needed to assess this hypothesis.

## Supplementary information

**Additional file 1: Figure S1.** Patient flow chart.

**Additional file 2: Figure S2.** Proportions of patients given epidural analgesia in each study ICU.

**Additional file 3: Table S1.** Sensitivity analysis restricted to the 526 patients in ICUs where at least 1 study patient received epidural analgesia.

**Additional file 4: Table S2.** Sensitivity analysis restricted to the 327 patients with an NRS pain score > 3 on ICU day 1.

## Data Availability

The dataset is available on reasonable request to the corresponding author.

## Abbreviations

95% CI: 95% confidence interval
AIS: Abbreviated Injury Scale
CT: Computed tomography
EA: Epidural analgesia
FRC: Functional residual capacity
ICU: Intensive care unit
ISS: Injury Severity Score
IMV: Invasive mechanical ventilation
NIV: Noninvasive ventilation
NRS: Numerical rating scale
NSAIDs: Nonsteroidal antiinflammatory drugs
RCT: Randomized controlled trial
SAPS II: Simplified Acute Physiology Score version II
VAP: Ventilator-associated pneumonia
