# Supplementary figures and images for: Epidural analgesia in ICU chest trauma patients with fractured ribs: retrospective study of pain control and intubation requirements
Source: Ann Intensive Care. 2020 Aug 27;10:116. doi: 10.1186/s13613-020-00733-0 (PMC7450151; doi:10.1186/s13613-020-00733-0)

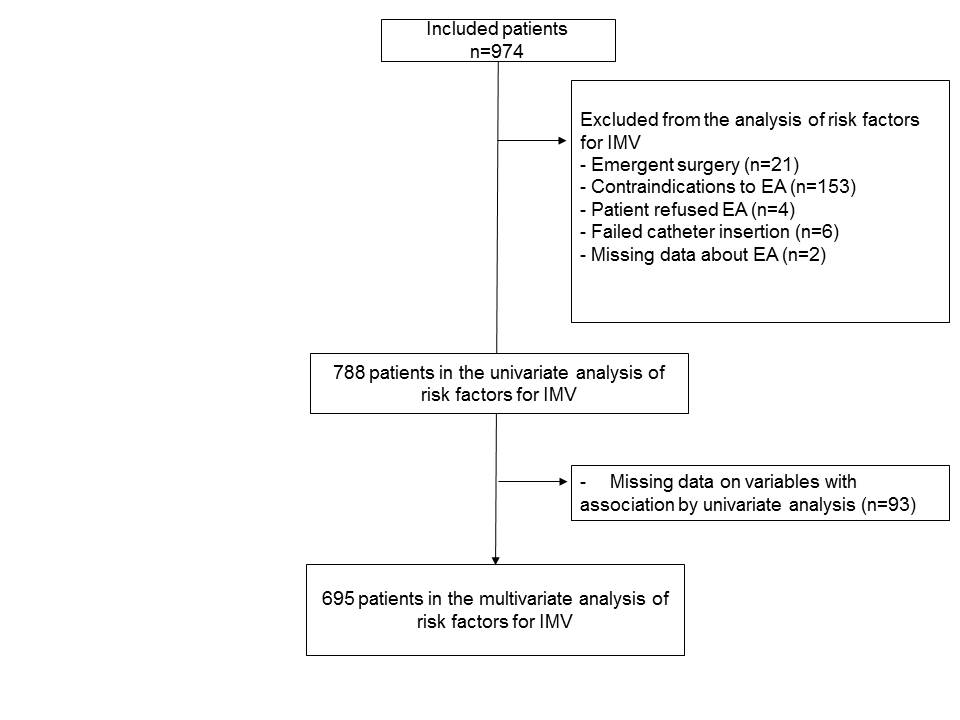

Supplement: Supplementary file 1 — Additional file 1: Figure S1. Patient flow chart. [file 13613_2020_733_MOESM1_ESM.jpg]
