# Supplementary material for: Epidural analgesia in ICU chest trauma patients with fractured ribs: retrospective study of pain control and intubation requirements
Source: Ann Intensive Care. 2020 Aug 27;10:116. doi: 10.1186/s13613-020-00733-0 (PMC7450151; doi:10.1186/s13613-020-00733-0)
Supplement: Supplementary file 2 — Additional file 2: Figure S2. Proportions of patients given epidural analgesia in each study ICU. [file 13613_2020_733_MOESM2_ESM.docx]

**Figure S2: Proportions of patients given epidural analgesia in each study ICU**
