# Supplementary material for: Epidural analgesia in ICU chest trauma patients with fractured ribs: retrospective study of pain control and intubation requirements
Source: Ann Intensive Care. 2020 Aug 27;10:116. doi: 10.1186/s13613-020-00733-0 (PMC7450151; doi:10.1186/s13613-020-00733-0)
Supplement: Supplementary file 3 — Additional file 3: Table S1. Sensitivity analysis restricted to the 526 patients in ICUs where at least 1 study patient received epidural analgesia. [file 13613_2020_733_MOESM3_ESM.docx]

| **Variable** | **HR** | **95%CI** | ***P* value** |
| --- | --- | --- | --- |
| **Alcohol abuse** |  |  |  |
| **With withdrawal syndrome** | **7.71** | **3.35 ; 17.7** | **<.0001** |
| Without withdrawal syndrome | 2.90 | 1.33 ; 6.32 | . |
| Respiratory rate at ICU admission | 1.05 | 0.99 ; 1.12 | 0.0963 |
| **SAPS II** | **1.07** | **1.05 ; 1.09** | **<.0001** |
| **Injury Severity Score** | **1.07** | **1.02 ; 1.12** | **0.0059** |
| Chronic respiratory disease | 2.10 | 0.97 ; 4.55 | 0.0613 |
| Thoracic EA | 0.57 | 0.25 ; 1.28 | 0.1721 |
| Noninvasive ventilation | 1.61 | 0.80 ; 3.25 | 0.1864 |
| **Flail chest** | **2.09** | **1.07 ; 4.10** | **0.0320** |

Table S1: Sensitivity analysis restricted to the 526 patients in ICUs where at least 1 study patient received epidural analgesia

HR, hazard ratio; 95%CI, 95% confidence interval; SAPS II, Simplified Acute Physiology Score version II; EA, epidural analgesia
