# Supplementary material for: Epidural analgesia in ICU chest trauma patients with fractured ribs: retrospective study of pain control and intubation requirements
Source: Ann Intensive Care. 2020 Aug 27;10:116. doi: 10.1186/s13613-020-00733-0 (PMC7450151; doi:10.1186/s13613-020-00733-0)
Supplement: Supplementary file 4 — Additional file 4: Table S2. Sensitivity analysis restricted to the 327 patients with an NRS pain score > 3 on ICU day 1. [file 13613_2020_733_MOESM4_ESM.docx]

| **Variable** | **HR** | **95%CI** | ***P* value** |
| --- | --- | --- | --- |
| **Alcohol abuse** |  |  |  |
| **With withdrawal syndrome** | **7.88** | **2.34 ; 26.5** | **0.0027** |
| Without withdrawal syndrome | 2.82 | 0.97 ; 8.17 | . |
| Respiratory rate at ICU admission | 1.05 | 0.98 ; 1.13 | 0.1903 |
| **SAPS II** | **1.06** | **1.03 ; 1.10** | **0.0006** |
| **Injury Severity Score** | **1.08** | **1.02 ; 1.14** | **0.0088** |
| **Chronic respiratory disease** | **3.13** | **1.27 ; 7.71** | **0.0130** |
| Thoracic EA | 0.43 | 0.14 ; 1.32 | 0.1378 |
| Noninvasive ventilation | 1.80 | 0.70 ; 4.61 | 0.2192 |
| **Flail chest** | **2.09** | **1.04 ; 6.91** | **0.0415** |

Table S2: Sensitivity analysis restricted to the 327 patients with an NRS pain score >3 on ICU day 1

HR, hazard ratio; 95%CI, 95% confidence interval; SAPS II, Simplified Acute Physiology Score version II; EA, epidural analgesia
